# Revaccination Response and Lack of Hepatitis B Reactivation After HCT for Sickle Cell Disease

**DOI:** 10.1111/tid.70097

**Published:** 2025-09-11

**Authors:** Henna Butt, Neal Jeffries, Triscia Martin, Valeria De Giorgi, Alison Zamora, John F. Tisdale, Matthew M. Hsieh

**Affiliations:** 1 Cellular and Molecular Therapeutics Branch, National Heart, Lung, and Blood Institute, National Institutes of Health, Bethesda, Maryland, USA; 2 Center for Cancer and Blood Disorders, Children’s National Hospital, Washington, DC, USA; 3 Office of Biostatistics Research, National Heart, Lung, and Blood Institute, National Institutes of Health, Bethesda, Maryland, USA; 4 Clinical Monitoring Research Program Directorate, Frederick National Laboratory for Cancer Research, Frederick, Maryland, USA; 5 Department of Transfusion Medicine, Clinical Center, National Institutes of Health, Bethesda, Maryland, USA

**Keywords:** HCT, hepatitis B, reactivation, sickle cell disease, vaccination

## Abstract

**Background:**

Sickle cell disease (SCD) can be cured by hematopoietic cell transplantation (HCT), but patients face increased risk of hepatitis B virus (HBV) reactivation due to immunosuppression. Understanding hepatitis B surface antibody (anti-HBs) kinetics is essential for optimizing HBV revaccination and posttransplant care.

**Methods:**

This post hoc analysis examined HBV immunity, reactivation, and revaccination response in 71 SCD patients who underwent HCT at the National Heart, Lung, and Blood Institute (2008–2021) using alemtuzumab and low-dose total body irradiation.

**Results:**

At baseline, 55% showed HBV immunity (anti-HBs ≥ 12 mIU/mL). Most patients responded to revaccination regardless of baseline immunity. Post-HCT revaccination was given to 93%, with 89% completing full series (Heplisav-B or Engerix-B). Vaccinated patients had a 67.5% chance of increased anti-HBs titers between Years 1 and 2, though no significant difference was seen compared to unvaccinated patients (*p* = 0.12). No HBV reactivation occurred; two patients with baseline HBcAb and HBsAg positivity showed decreasing HBV DNA levels.

**Conclusions:**

Results indicate that HBV immunity can decline post-HCT, but most patients remain immune, and revaccination is effective. However, some non-responders—especially those treated with IVIG, rituximab, or prolonged immunosuppression—need further study. Prospective research is needed to optimize revaccination timing and immune monitoring in this high-risk group.

## 1 | Introduction

Sickle cell disease (SCD) affects millions of individuals worldwide [1]. It is the result of a monogenic disorder in which a mutation in the beta globin gene (*HBB*) causes a change in the shape of red blood cells resulting in sickle-shaped erythrocytes [2]. These cells are rigid leading to vessel occlusion and hemolytic anemia. The result is organ damage and significant morbidity and mortality [3]. While disease modifying agents such as hydroxyurea [4] exist to manage symptoms of the disease, the only cure for SCD is hematopoietic cell transplant (HCT) [5].

Hepatitis B virus (HBV) infection is a significant global health concern, affecting millions of individuals worldwide [6]. Among patients with SCD undergoing HCT, managing HBV infection is particularly challenging due to their immunocompromised state and the risk of viral reactivation posttransplantation [7]. Chemotherapy, lymphodepletion as part of the conditioning regimen, and post-HCT immunosuppression exacerbate this risk, potentially leading to severe consequences for HBV carrier recipients [8]. One critical factor in this context is the persistence and waning of hepatitis B surface antibody (anti-HBs) titers, which are pivotal in providing immunity against HBV. However, there is a gap in current literature documenting the dynamics of anti-HBs titers in SCD patients post-HCT, especially during the first year following transplantation.

Undergoing allogeneic HCT complicates the infectious course of HBV, potentially leading to hepatic failure, chronic active hepatitis, or cirrhosis [9]. Patients who are positive for hepatitis B core antibody (HBcAb) prior to HCT have another layer of complexity in their post-HCT care. HBcAb positivity indicates previous HBV exposure, placing individuals at a heightened risk for reactivation during post-HCT immunosuppression [10, 11]. Reactivation can occur 10–48 months post-HCT, often triggered by chemotherapy in conditioning regimens [12, 13] and clinical exacerbation occurs in approximately 60% of cases within the first year [14]. While most patients recover and achieve HBsAg negativity, a small proportion (3%) progress to fulminant hepatic failure [9]. Reactivation is particularly challenging to treat in the peri-transplant period due to altered host immune responses [15].

Although studies on non-SCD populations indicate that monitoring HBV serology, ALT, and HBV DNA can facilitate early detection of exacerbations, this has not been well-studied in SCD patients. Reverse seroconversion and reactivation have been observed in patients with occult or resolved HBV infections, even in those undergoing prophylactic antiviral treatment [16–18].

Most patients lose anti-HBs during conditioning and require post-HCT revaccination. Current guidelines recommend initiating HBV revaccination after 12 months, aligning with expected immune recovery. Nonetheless, uniform recommendations across diverse HCT indications may not account for the unique immune reconstitution patterns in SCD patients [19] or the heterogeneity of conditioning regimens. Studies report a seroconversion rate of 40% after HBV revaccination, with responses improving to 82% after a complete series [7, 20]. However, response rates remain suboptimal in patients who were anti-HBs seronegative pre-HCT, highlighting the need for tailored revaccination strategies. Furthermore, post-HCT revaccination has been shown to lower rates of HBV reactivation [10].

While the interplay between HBV and HCT has been extensively studied in general populations, its implications for SCD patients remain underexplored. Understanding the kinetics of anti-HBs titers in this specific patient population is critical. As curative HCT options become more accessible for SCD, addressing these gaps is essential for optimizing revaccination strategies and management in this vulnerable group.

## 2 | Methods

### 2.1 | Study Design

This study is an IRB-approved post hoc analysis evaluating HBV reactivation and response to revaccination in patients with SCD undergoing HCT. The study includes SCD patients who have undergone matched related donor HCT with documented HBV status both before and after transplantation. Patients with SCD and banked specimens who underwent HCT at the National Heart, Lung, and Blood Institute (NHLBI) under IRB-approved protocols 03-H-0170 (clinicaltrials.gov NCT00061568) and 14-H-0077 (NCT002105788) between 2008 and 2021 were included (*N* = 71). Pre-HCT conditioning regimens included alemtuzumab totaled 1 mg/kg and 300 cGy total body irradiation. Protocol 14-H-0077 included two additional chemotherapy agents (Table 1) and was more immunodepleting than 03-H-0170. Patients were excluded from analysis if they had an inadequate number of available serum samples for serological assessment.

### 2.2 | Data Collection

Data collection involved comprehensive HBV serological assessments, including baseline HBV serology and annual follow-ups measuring HBsAg, HBs antibody, HBc IgM antibody and total HBc antibody, and HBV DNA levels. Post-HCT monitoring focused on HBV reactivation, the timing of HBV revaccination, and immune response evaluation based on anti-HBs antibody titers. Additional data points included chimerism levels, the presence of graft-versus-host disease (GVHD), HBV prophylactic anti-viral medication use, and liver function tests (LFTs). The study also assessed parameters such as time to engraftment, IVIG dosing, duration of immunosuppression, and occurrence of graft failure. When available, liver biopsy results, liver iron concentration (LIC), corticosteroid use, and rituximab dosing were documented to provide further insights into patient outcomes. The recombinant hepatitis B vaccine series included two doses of 20 mcg of adjuvanted Heplisav-B, at 1 and 1.5 years given to adults, or three doses of 10 mcg of nonadjuvanted Engerix-B, at 1, 1.5, and 2 years given to pediatric patients. Patients who were HBcAb positive received lamivudine or entecavir as per our institutional hepatitis viral prophylaxis guidelines.

### 2.3 | Statistical Analysis

The binomial distribution function was used to assess whether the proportion of individuals with decreasing titers between baseline and 1 year follow-up was significantly different from 50%. This was separately assessed for those with positive titer levels at baseline (anti-HBs titers ≥ 12 mIU/mL) and those with negative titer levels (< 12 mIU/mL). A chi-square test was used to test whether the proportions with decreasing titers differed between the groups with positive and negative baseline titer levels. The binomial distribution was also used to assess whether the proportion of individuals with decreasing titers between 1 and 2 years was significantly different from 50% in the subgroup that received at least one revaccination at either 1 or 1.5 years post-HCT. Fisher’s exact test was used to test whether the proportions with decreasing titers differed between the groups with and without at least one revaccination at 1 or 1.5 years post-HCT. Fisher’s test was used instead of a chi-square test because the sample size of the group without at least one revaccination was small (*N* = 5). Chi-square or Fisher’s tests (depending upon the sparsity of data) were also used to assess associations between titer levels following revaccination and the potential explanatory factors of protocol type, HLA allele characteristics, CD3+ cell counts, and CD19+ cell counts. R version 4.3.2 was used for analyses.

Changes in myeloid and lymphoid chimerism following a decline in anti-HBs titer protection were examined visually and were generally seen to mirror the typical patterns of decline in myeloid chimerism and increase in lymphoid chimerism following HCT.

## 3 | Results

### 3.1 | Demographic Data

Most patients (66%) were treated on 03-H-0170 while 34% were treated on 14-H-0077. A total of 27 patients (38%) received red cell transfusions outside of the United States prior to HCT. There two patients who had acute GVHD (one Grade 2 skin: treated with topical steroids; one Grade 3 lower GI: treated with systemic steroids). There was one patient with chronic GVHD (mild to moderate with dry eyes and dry mouth). Twelve patients were treated with HBV prophylaxis entecavir or lamivudine based on HBcAb positivity. Eleven (15%) patients received IVIG treatment in the peri-transplant period. Patients received immunosuppression for a median of 14 months post-HCT. There were five (7%) patients who experienced graft failure, one of whom developed acute myeloid leukemia (AML). The median ALT and AST values respectively were 27.5 and 36.5 U/L pre-HCT. The median time to neutrophil and platelet engraftment was 22 and 20 days, respectively. The median myeloid (CD14/15) and T cell (CD3) chimerism levels at 1–3 years post-HCT and key demographic data are included in Table 1.

### 3.2 | Baseline Immunity to HBV

**FIGURE 1 |:**
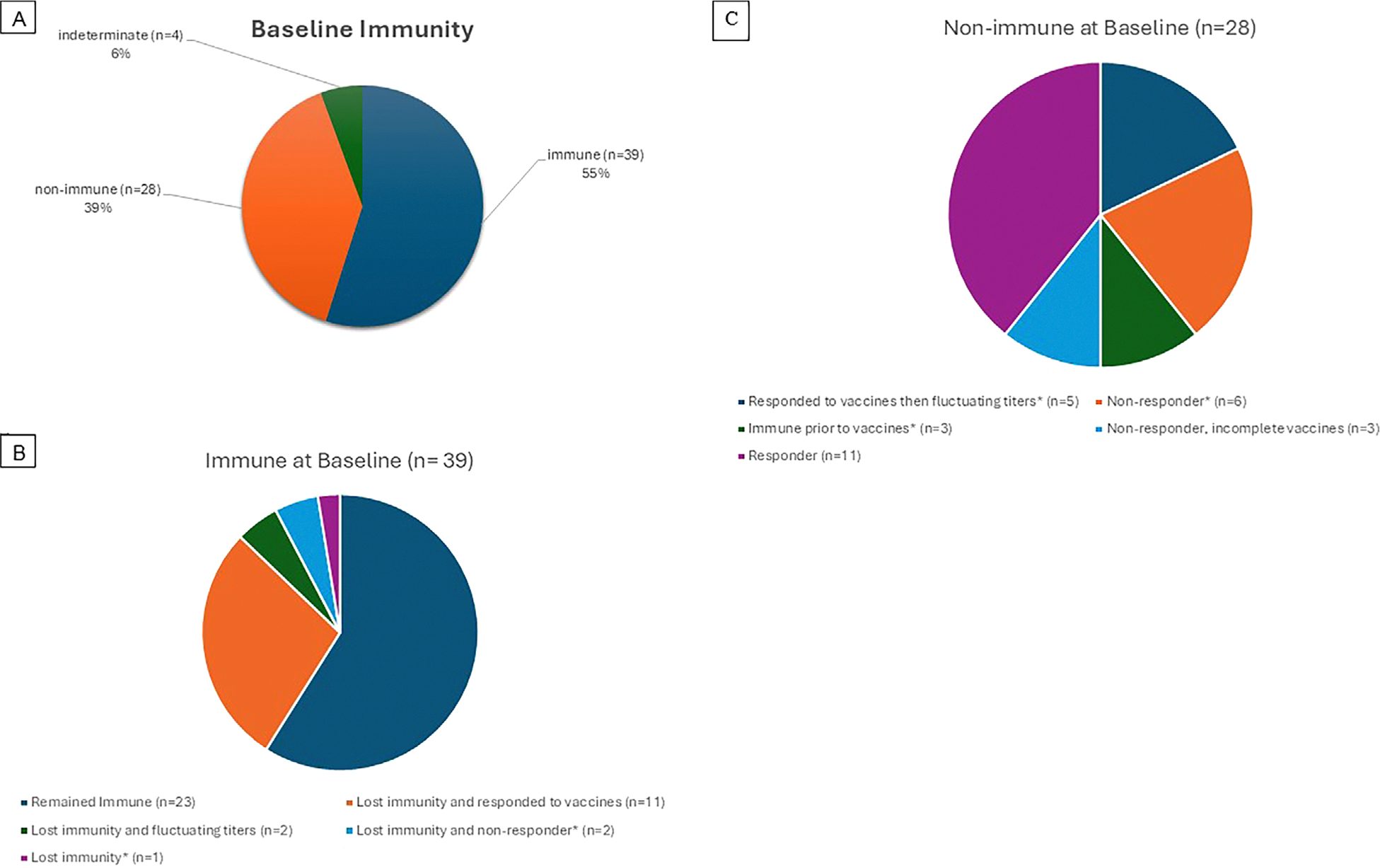
Pie charts reflecting immunity to HBV and outcomes. (A) Overall baseline immunity status of the cohort. (B) Outcomes in patients who were immune to HBV at baseline. (C) Outcomes in patients who were non-immune to HBV at baseline.

A total of 39 (55%) patients had baseline immunity to HBV as evidenced by positive anti-HBs titers ≥ 12 mIU/mL (Figure 1A). Furthermore, 16 had HBs titers ≥ 100 mIU/mL indicating a strong immune response and high degree of immunity. Among those who were immune at baseline, 28 individuals remained immune and 11 had titers that declined to a non-immune range (Figure 1B). The proportion of decreasing titers at 1 year post-HCT was 81% for those with immune baseline levels and 67% for those with non-immune baseline levels (*p* value for equivalent proportions 0.44). These results indicated titers waned in the first year following HCT regardless of baseline immunity status. In addition, patients who were immune at baseline were more likely to retain immunity post-HCT (Figure 1B).

### 3.3 | Revaccination Response

**FIGURE 2 |:**
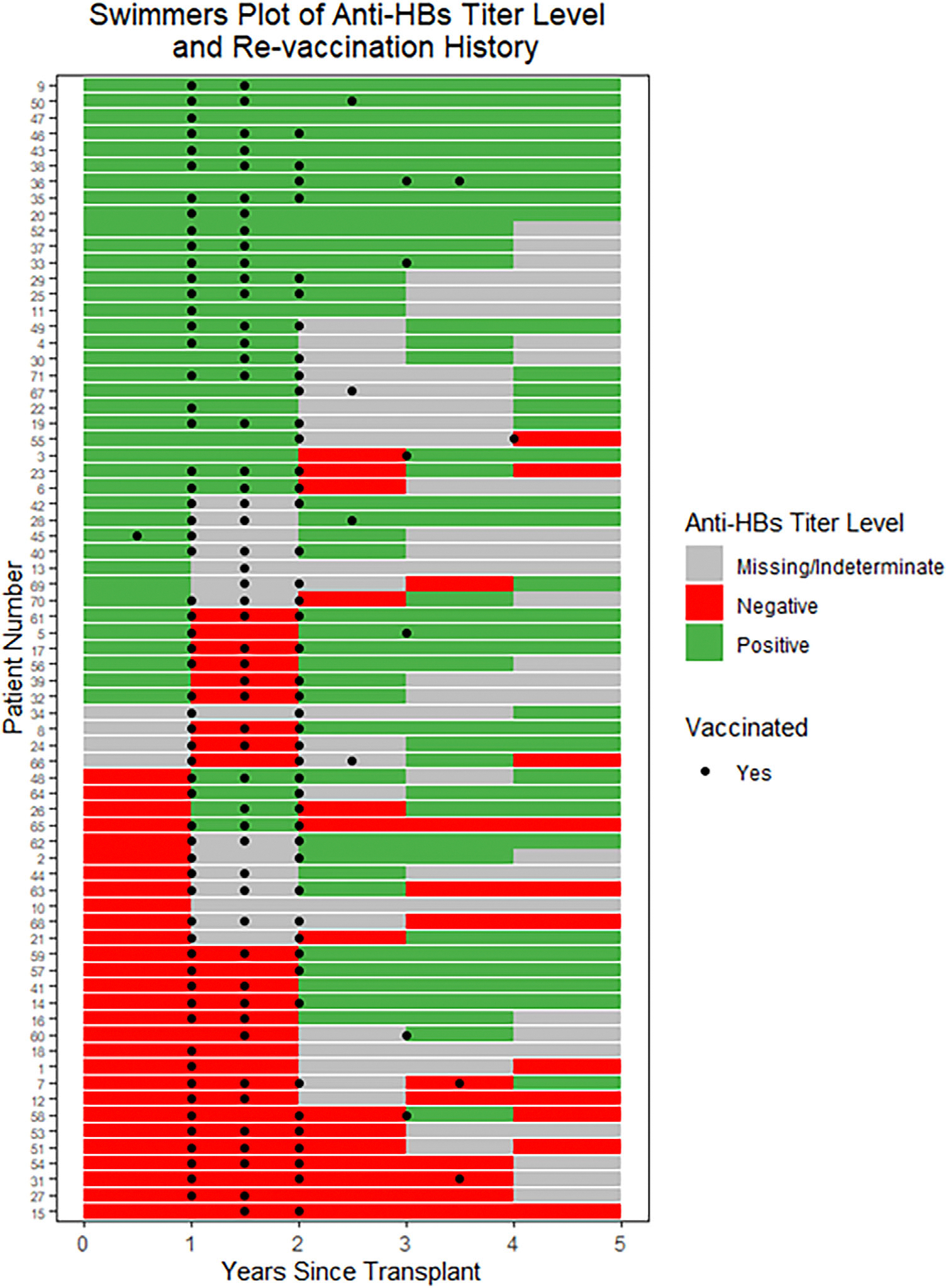
Swimmers plot depicting individual patient immunity status as indicated by anti-HBs titers from baseline to post hematopoietic cell transplant follow-up visits, with vaccination timings overlaid. Each row represents a single patient, illustrating changes in HBV immunity over time. The color bar for a year of time (e.g. Years 0–1) corresponds to the anti-HBs titer level at the beginning of the year. Green bars represent immunity, red bars represent non-immunity, grey bars represent indeterminate or missing data, while black circles indicate timing of revaccination.

A total of 93% of the patients were vaccinated starting at 1 or 1.5 years post-HCT and a total of 89% received a full revaccination series (two doses of Heplisav-B or three doses of Engerix-B). The mean number of vaccines required to achieve response was 2 (Figure 2). There were similar degrees of baseline immunity between both protocols; 67% for 14-H-0077 and 49% for 03-H-0170 (*p* = 0.24). Following a complete vaccine series, anti-HBsAb titer responses above 12mIU/mL were comparable across both protocols at 78% for 14-H-0077 and 77% for 03-H-0170.

We examined revaccination response in several ways. First, among patients vaccinated at 1 or 1.5 years post-HCT, 70% showed increasing titers between Years 1 and 2 (*p* = 0.02, for the null hypothesis that the true proportion is 50%). Notably, most patients responded to vaccines regardless of baseline immunity to HBV (Figure 1B,C). Comparing the probabilities of increasing titers between the vaccinated and unvaccinated groups, there was no significant difference (*p* = 0.11). However, this result may be influenced by the small sample size of the baseline unvaccinated group (*N* = 5). Second, when we analyzed the proportion of patients who responded to a complete set of revaccination, there were 36 of the 39 individuals who were immune at baseline, compared to 19 of 25 who were non-immune at baseline (*p* = 0.14). Third, we analyzed the magnitude of anti-HBs titer increase, the median and mean values of increase between immune at baseline versus non-immune at baseline were 33.3 and 2002.8 versus 34.9 and 603, respectively, *p* = 0.63. Restricting to those who responded in both groups, median and mean values of anti-HBs increase were 81.8 and 2185.9 versus 75.8 and 854.1, respectively, *p* = 0.48. Finally, we evaluated revaccination response in association with the presence of HLA alleles DRB1–03, DRB1–07, or DQB1–02. Those with or without these HLA alleles responded similarly in terms of anti-HBsAb titer levels above 12 mIU/mL following revaccination, 71% versus 82% (*p* = 0.51).

There were increases in anti-HBs prior to revaccination or in the unvaccinated individuals, which could reflect donor immunity. The anti-HBs assay could not differentiate between donor and patient antibodies. Natural infection was ruled out by no new detection of anti-HBc and HBsAg. Another source of these antibodies could have been red blood cell transfusions; however most patients received red cell or platelet transfusions during the first 30 days post HCT, making this less likely. There was no association between decreasing titer levels following revaccination and chimerism levels. Among the nine vaccine non-responders, one patient had graft failure and received IVIG, two patients received rituximab, and five patients received prolonged immunosuppression detailed in Tables S1 and S2.

### 3.4 | Lack of HBV Reactivation

HBV reactivation was defined as HBsAg > 5 IU and total anti-HBc < 0.9 IU. There were 12 patients at risk who were anti-HBc positive at baseline. Of those, two patients had HbsAg levels that were positive. One patient had HBV DNA levels < 116 copies at 1 year post-HCT. The other patient had fluctuating levels ranging from < 20 to 3195 copies from baseline to 10 years post-HCT. Hepatitis B vaccination records for these two patients could not be verified prior to HCT. Both received entecavir prophylaxis starting at the HCT conditioning through 1 year post-HCT. The remaining 10 patients who were HbcAb positive at baseline had negative HbsAg levels and HBV DNA levels that were undetectable or not quantifiable throughout the post-HCT time points. Overall there were no HBV reactivation in this subgroup of patients.

### 3.5 | Immune Reconstitution

To evaluate functional immune responses which could provide deeper insight into immune protection we also examined absolute CD3+ (T cell) and CD19+ (B cell) counts at 1 year post-HCT and anti-HBs titer levels following revaccination. We used two different thresholds (200 and 1000 cells/μL for CD3+ cells and 20 and 100 cells/μL for CD19+). There were no patients with CD3+ cell counts < 200 cells/μL. Using a higher threshold for more clinical relevance, those with CD3+ <1000 cells/μL (*N* = 24) had a similar distribution of post-revaccination titers below the threshold compared to those with CD3+≥ 1000 cells/μL (*p* = 0.36). Among patients with B cell counts measured at 1 year, there was one patient with B cell count < 20 cells/μL and 43 patients with B cell count ≥ 20 cells/μL who had a positive immune response after revaccination (*p* = 1.00). It should be noted that the low B cell group had a very small sample. For B cell counts < 100 cells/μL at 1 year, only three patients’ counts fell below the threshold, and the distribution of titer responses also did not differ significantly from those with B cell counts > 100 cells/μL (*p* = 1.00).

## 4 | Discussion

Our study provides insights into HBV immunity, revaccination response, and lack of HBV reactivation in patients with SCD who underwent HCT. HBV immunity at baseline was present in 55% of patients. Over the first year post-HCT, a decrease in titers was observed in patients who were immune at baseline. The observed decline in anti-HBs titers is expected since our patients underwent conditioning and remained on immunosuppression for 12–15 months post-HCT. These two low-intensity HCT regimens have higher graft failure rates of 6%–10% compared to fully myeloablative regimens (< 2%), thus justifying the longer duration immunosuppression in the absence of GVHD. This reduction in anti-HBs titer posttransplantation aligns with existing literature suggesting that immune memory can wane after intensive conditioning and immunosuppressive therapy in the HCT and solid organ setting [21]. Importantly, while titers did wane, most patients who were immune at baseline remained immune post-HCT.

The observed probability of increasing titers in re-vaccinated individuals between Years 1 and 2 post-HCT was 67.5%, consistent with the literature reporting post-revaccination seroconversion [22–24]. A majority of patients responded to vaccines regardless of baseline immune status. However, a significant difference was not observed between vaccinated and unvaccinated groups (*p* = 0.11) suggesting that additional factors, such as small sample size (*N* = 5) and individual immune reconstitution variability influenced vaccine efficacy. In addition, there was no observed correlation between chimerism levels and changes in titer responses post-revaccination.

Several notable clinical factors were identified upon examination of the nine vaccine nonresponders. These included graft failure, IVIG administration, rituximab use, and prolonged immunosuppression use, all of which could impact immune response, consistent with literature in the solid organ transplant setting [23]. Immunosuppressive agents can impair immune function to varying degrees, showing a specific link between their use and decreased vaccine effectiveness due to impaired B-cell immunity [24]. Rituximab treatment has been associated with increased risk of revaccination titer loss posttransplant [25]. This finding underscores the importance of individualized revaccination strategies and further investigation into potential predictors of vaccine nonresponsiveness [21]. A total of 59 patients continued to receive immunosuppressive drugs at 1 year or longer post-HCT, which is when we began the hepatitis B revaccination series. Since the effects on lymphocyte function can last weeks or months after stopping immunosuppression, it is likely that the effects of immunosuppression were present during the entire period of hepatitis B revaccination.

Our analysis of CD3+ and CD19+ cell counts at 1 year post-HCT aimed to explore the association between immune cell recovery and functional vaccine responses. While presence of these cells are often used as surrogates for immune competence, our findings indicate that most patients had sufficient CD3+ and CD19+ cell counts at 1 year, and suggest there was no impaired humoral response to revaccination. However, these findings should be interpreted with caution due to limited sample sizes, particularly in groups with low cell counts. In addition, these may not well represent immune reconstitution necessary for response to revaccination. Future studies should aim to evaluate functional assessments such as T-cell proliferation or immunoglobulin levels.

Nonresponsiveness to HBV revaccination has been partly attributed to genetic factors, particularly autosomal dominant expression of certain HLA Class II alleles, including HLA-DRB103, HLA-DRB107, and HLA-DQB1*02 [26–28]. In line with previous studies, 29% of our cohort did not respond to first-dose vaccine, and this proportion was not different than those without these HLA alleles. The limited sample size may reduce the generalizability of this finding.

There were 12 patients at potential risk of reactivation, with two patients at higher risk, denoted by positive HBsAg levels and measurable HBV DNA copies pre-HCT. Both patients had undetectable anti-HBs and received entecavir prophylaxis prior to conditioning regimen and for the 1 year post-HCT. Neither had substantial increase in HBV DNA copies to suggest a flare or recrudescence. The other 10 patients had no detectable HBV DNA in the post-HCT time points. The lack of reactivation in this cohort indicated that prophylactic antiviral therapy and vigilant monitoring were successfully implemented. This outcome underscores the importance of adhering to established guidelines for the prevention and management of HBV reactivation in at-risk patients.

This study has limitations. First, its retrospective design limits the ability to establish causality between HCT, HBV immunity, and reactivation, while reliance on existing medical records may introduce data inconsistencies. In addition, the study was conducted at a single institution, potentially limiting the generalizability of findings to other centers with different HCT protocols. The small sample size, particularly in subgroup analyses such as vaccinated versus unvaccinated patients, likely reduced statistical power, making it difficult to draw definitive conclusions when tests fail to show statistical significance. Because of the small sample size there may in fact be clinically meaningful differences between compared subgroups but not enough subjects to statistically demonstrate this and larger sample sizes are necessary to clarify such ambiguity. Variability in revaccination timing further complicates interpretation of immune responses. Selection bias may also be present, as only patients with available banked serum samples were included, potentially limiting representation of the broader SCD-HCT population.

Overall, our findings highlight the complex interplay between HCT, immune reconstitution, and HBV immunity. While revaccination appears to be effective for most patients, a subset remains at risk for inadequate immune responses, necessitating ongoing surveillance. Future prospective studies with larger, multi-center cohorts and standardized follow-up protocols are needed.

## Supplementary Material

supp table 1

supp table 2

visual abstract

Supporting Information

Additional supporting information can be found online in the Supporting Information section.

**Supporting Table 1:** tid70097-sup-0001-tableS1.docx. **Supporting Table 2:** tid70097-sup-0002-tableS2.docx. **Supporting File1:** tid70097-sup-0003-VisualAbstract.pdf.

## Figures and Tables

**TABLE 1 |:** Demographic data.

Variable	03-H-0170 Alemtuzumab TBI300 cGy Sirolimus *N* = 47	14-H-0077 4 doses of IV pentostatin Oral cyclophosphamide Alemtuzumab TBI 300 cGy Sirolimus *N* = 24

Age median (min, max)	31 (10, 64)	28.5 (10, 45)
Female gender - *N* (%)	11 (23%)	12 (50%)
Race		
Asian	0 (0%)	1 (4%)
Black/African American	44 (94%)	21 (88%)
Other	0 (0%)	1 (4%)
Multiracial	2 (4%)	1 (4%)
White	1 (2%)	0 (0%)
Ethnicity		
Hispanic or Latino	1 (2%)	0 (0%)
Not Hispanic or Latino	46 (98%)	21 (88%)
Other	0 (0%)	1 (4%)
Unknown	0 (0%)	2 (8%)
Genotype		
Beta Thal Major	0 (0%)	1 (4%)
S B+	1 (2%)	1 (4%)
S B0	5 (11%)	0 (0%)
SC	2 (4%)	0 (0%)
SS	39 (83%)	22 (92%)
Received red cell transfusions outside of the United States	17 (36%)	10 (37%)
GVHD	0 (0%)	acute: 2 (8%), 1Gr2 skin, 1Gr3 lower GI Chronic: 1 (4%) mild to moderate (eye, mouth)
HBV prophylaxis		
Entecavir	3 (6%)	1 (4%)
Lamivudine	6 (13%)	3 (12%)
None	38 (81%)	20 (83%)
IVIG doses (number)		
0	40 (85%)	20 (83%)
1	1 (2%)	0 (0%)
2	0 (0%)	3 (12%)
3	2 (4%)	1 (4%)
5+	4 (9%)	0 (0%)
Immunosuppression duration (months) Median (min, max)	15 (6, 60)	12 (2, 46)
Graft failure – *N* (%)	3 (6%)	2 (8%)
ALT (U/L) *N*; median (min, max)		
Pretransplant	47; 27 (14, 134)	23; 28 (8, 104)
1 year posttransplant	42; 37.5 (15,104)	19; 32 (8, 74)
2 years posttransplant	33; 28 (1, 85)	17; 22 (12, 55)
3 years posttransplant	36; 20.5 (10, 51)	10; 18.5 (7, 104)
AST (U/L) *N*; median (min, max)		
Pretransplant	47; 34 (13,139)	23; 43 (16, 114)
1 year posttransplant	42; 34 (15, 92)	19; 39 (15, 90)
2 years posttransplant	33; 27 (1, 77)	17; 26 (13, 51)
3 years posttransplant	36; 24 (10, 37)	14; 23 (12, 73)
Time to neutrophil engraftment (days)	40; 21.5 (7, 37)	21; 22 (8, 67)
N; median (min, max)		
Myeloid chimerism (%)		
*N*; median (min, max)		
1 year posttransplant	41; 99 (0, 100)	17; 100 (0,100)
2 years posttransplant	33; 99 (10,100)	16; 99 (44, 100)
3 years posttransplant	36; 98 (0, 100)	14; 99 (44,100)
Lymphoid chimerism (%)		
*N*; median (min, max)		
1 year posttransplant	41; 55 (0, 100)	17; 61 (0,100)
2 years posttransplant	33; 67 (10,100)	16;62 (27, 98)
3 years posttransplant	36; 71 (0, 84)	14; 61.5 (0, 99)

Abbreviations: ALT, alanine transaminase; AST, aspartate aminotransferase; GVHD, graft versus host disease; HBV, hepatitis B virus; IVIG, intravenous immunoglobulin.

## Abbreviations:

ALT: alanine aminotransferase
AML: acute myeloid leukemia
anti-HBs: hepatitis B surface antibody
AST: aspartate aminotransferase
GVHD: graft versus host disease
HBcAb: hepatitis B core antibody
HBsAg: hepatitis B surface antigen
HBV: hepatitis B virus
HCT: hematopoietic cell transplant
IVIG: intravenous immune globulin
LFT: liver function test
LIC: liver iron concentration
SCD: sickle cell disease
