# Supplementary material for: Revaccination Response and Lack of Hepatitis B Reactivation After HCT for Sickle Cell Disease
Source: Transpl Infect Dis. 2025 Sep 11;27(6):e70097. doi: 10.1111/tid.70097 (PMC12720196; doi:10.1111/tid.70097)
Supplement: Supplementary file 1 — Supporting Table 1: Clinical information for patients who were at immune for HBV at baseline and HBV vaccine unexpected outcomes. [file TID-27-e70097-s001.docx]

**Supplemental Table 1.** Clinical information for patients who were at immune for HBV at baseline and HBV vaccine unexpected outcomes.

| **Immune at baseline** | **Course** | **Possible explanation (s)** |
| --- | --- | --- |
| Lost immunity + non responder (n=3) | 1. 32 year old male vaccinated at years 1, 1.5 and 2 and was immune until year 1.5 then non-immune at 2 and 5 years. | Received lamivudine prophylaxis and had prolonged immunosuppression. |
|  | 2. 28 year old male vaccinated at years 2 and 4, was immune until year 1 then non-immune at years 2.5 and 4. | Received lamivudine prophylaxis and had prolonged immunosuppression. |
|  | 3. 39 year old female vaccinated at years 1, 1.5 and 2, remained immune until year 5 then became non-immune. | Received lamivudine, rituximab and IVIG. |
