# Supplementary material for: Revaccination Response and Lack of Hepatitis B Reactivation After HCT for Sickle Cell Disease
Source: Transpl Infect Dis. 2025 Sep 11;27(6):e70097. doi: 10.1111/tid.70097 (PMC12720196; doi:10.1111/tid.70097)
Supplement: Supplementary file 2 — Supporting Table 2: Clinical information for patients who were at non‐immune for HBV at baseline and HBV vaccine unexpected outcomes. [file TID-27-e70097-s003.docx]

**Supplemental Table 2.** Clinical information for patients who were at non-immune for HBV at baseline and HBV vaccine unexpected outcomes.

| **Non-immune at baseline** | **Course** | **Possible Explanation(s)** |
| --- | --- | --- |
| Responded then fluctuating titers (n=5) | 1. 28 year old female vaccinated at years 1 and 2. Non-immune until year 3, remained immune from 3-5, then non-immune from years 6-7. | Prolonged immunosuppression |
|  | 1. 34 year old female vaccinated at 1, 1.5 and 2 years (3 vaccines), immune at 1.5 year then non-immune from years 2-5. | Received biologic treatment and corticosteroids for Rheumatoid arthritis and prolonged immunosuppression |
|  | 3. 53 year old male vaccinated at year 1, 1.5 and 2 years, immune at year 2 then non-immune at years 3-4. | Received several courses of anti-B lymphocyte treatments (corticosteroids, IVIg, rituximab, bortezmib) for red cell antibody and prolonged immunosuppression |
|  | 4. 41 year old male vaccinated at 1,2 and 3 years, immune at year 3 then non-immune at year 4. | Prolonged immunosuppression |
|  | 5. 35 year old male vaccinated at 1, 1.5 and 2 years. Immune at 1-2 years. Lost immunity years 3-4 and regained at year 5. |  |
| Immune prior to vaccines (n=3) | All patients received 2-3 vaccinations after 1 year, all were immune at 1 year (prior to vaccines) and all remained immune through year 5Ages 21-35 | 1 patient with low titers throughout (<100 mIU/mL).  1 patient with low titers which then improved (>100 mIU/mL) after vaccinations.  1 patients with fluctuations from high to low titers. |
| Non-responder (n=6) | All received 2-3 vaccines from years 1-2. Remained non-immune through follow-up ranging from 2-10 years post HCT. Ages 18-45 | 1 patient with graft failure and received IVIG.  Two patients received rituximab.  One patient received entecavir prophylaxis. Two patients were on prolonged immunosuppression. |
