# Supplementary material for: Revaccination Response and Lack of Hepatitis B Reactivation After HCT for Sickle Cell Disease
Source: Transpl Infect Dis. 2025 Sep 11;27(6):e70097. doi: 10.1111/tid.70097 (PMC12720196; doi:10.1111/tid.70097)
Supplement: Supplementary file 3 — Supporting File 1: tid70097‐sup‐0003‐VisualAbstract.pdf. [file TID-27-e70097-s002.pdf]

Re-vaccination Response and Lack of Hepatitis B Reactivation after HCT for SCD

@TheTxIDJournal

Butt, H *et al*. ***Transplant Infectious Diseases***. 2025.

Background:

- Sickle cell disease (SCD) can be cured by hematopoietic cell transplantation (HCT)
- Patients face increased risk of hepatitis B virus (HBV) reactivation due to conditioning regimen and immunosuppression.
- Understanding hepatitis B surface antibody kinetics is essential for HBV re-vaccination and post-HCT care.

Results:

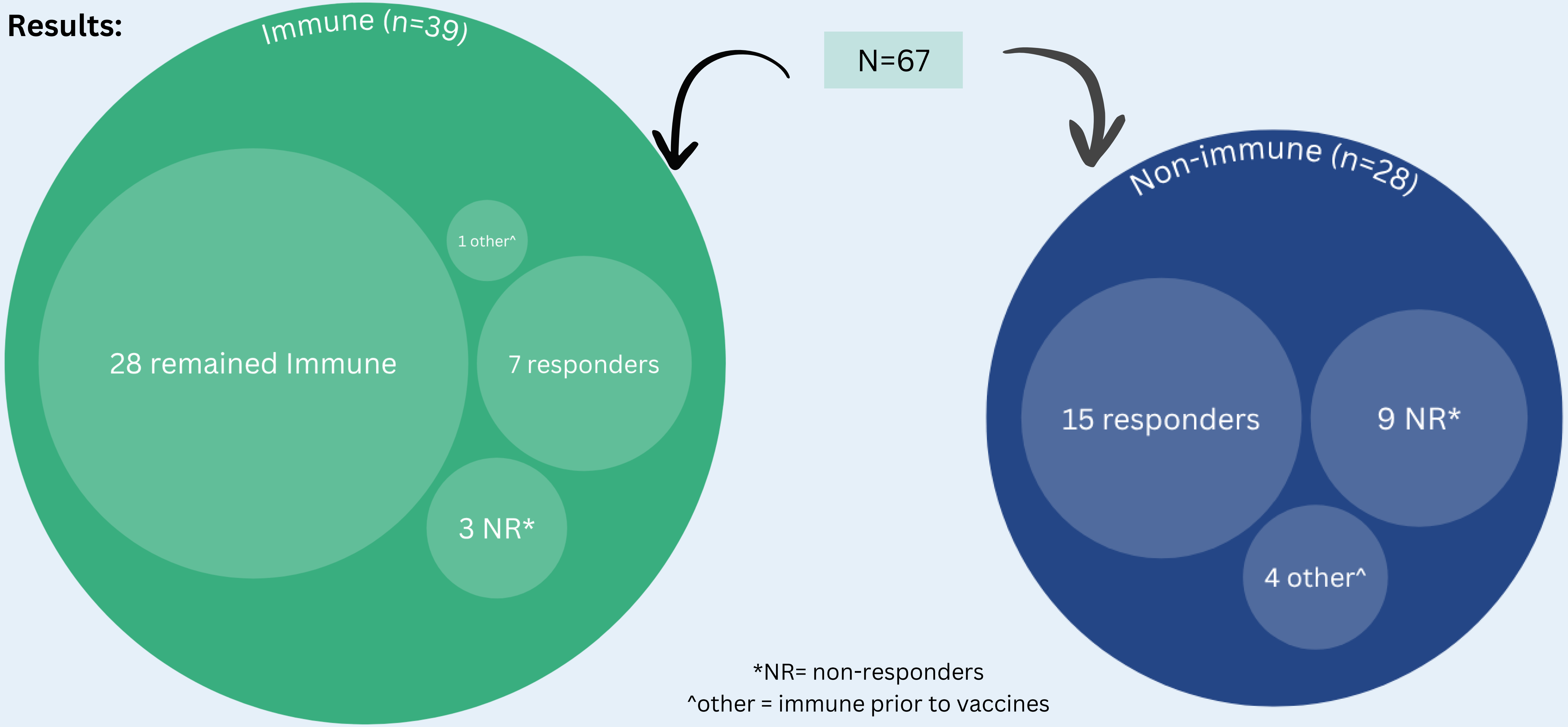

*NR= non-responders
^other = immune prior to vaccines

Methods:

Post-hoc analysis

Patients with SCD who underwent HCT at the NHLBI between 2008-2021

HBcAb, HBsAg, HBsAb, HBV DNA samples pre-HCT and 1-5 years post-HCT time points

Conclusions:

Most remained immune or responded to HBV titers revaccination post-HCT

Non-responders to re-vaccination had underlying clinical factors

No HBV reactivation occurred
